# Income level and healthcare utilization in Calabar Metropolis of Cross River State, Nigeria

**DOI:** 10.1016/j.heliyon.2020.e04983

**Published:** 2020-09-28

**Authors:** Esther P. Archibong, Glory E. Bassey, Brown E. Isokon, Rosemary Eneji

**Affiliations:** a Department of Social Work, University of Calabar, Nigeria; b Department of Sociology, University of Calabar, Nigeria

**Keywords:** Income, Utilization, Healthcare, Services, Socioeconomic, Community, Economics, Sociology, Public health

## Abstract

Throughout the world, socio-economic status differentials of individuals have a lot of influence on privileges and opportunities enjoyed by society members. The different social and economic positions of individuals and income levels oftentimes hinder them from effective use of health care facilities. It is against this background, that the study was designed to investigate income differentials and its influence on utilization of healthcare services among the people of Calabar Metropolis in Cross River State, Nigeria. Relevant body of literature were reviewed and survey design method was adopted. The instruments used were the questionnaire, interview and Focus Group Discussions (FGD). A sample of 360 respondents was selected using a multi-stage sampling procedure for the study. Data was presented using simple percentage calculation while Chi-Square and ANOVA statistical tool was used to test and ascertain the relationship between income level and healthcare services usage at 0.05 level of significance. Findings of the study revealed that level of income and occupational status of individual influence health care service utilization among people in Calabar Metropolis. At the end of the study, several recommendations were made among which were a general improvement of socio-economic status of the people in areas of availability and compulsory education, employment opportunities as well as equitable distribution of income and minimizing cost of health care services for effective and equal utilization.

## 1 Introduction

Everywhere in the world, there exist copious debates on the roles that socio-economic status differential of people plays on the degree of health-seeking behavior, and data about the usage of healthcare facilities to help their general health condition. This issue is troubling especially in Sub-Saharan African with 70 percent of the population of low socio-economic status, and are mostly found in rural communities as well as confined to the informal sector of the economy (Buor, 2005). According to Anderson (2016), the choice of utilizing health services involves ability to perceive and recognize symptoms, the degree to which the side effects are seen as hazardous, the measure of resilience for the manifestations, and essential needs that lead to rejection of health services utilization. Several factors such as cultural, social, gender, economic and geographic variables predispose people to poor utilization of health services. The dimensions normally connected with socioeconomic status differentials are occupational status, educational accomplishment, income, poverty and wealth (Krieger et al., 1997).

In numerous societies, there are individuals and groups placed in prominent economic status having better privileges and opportunities to enjoy better health care services than their low income counterparts. Abodurin (2010), added that the decision to utilize a particular health care facility is to a great extent controlled by the satisfaction derived from services and the apparent nature of care provided. This decision is sometimes restricted by variables for example, affordability, accessibility, availability of services of the health care outlets; customs and beliefs, critical nature of care required including the confidence in the efficacy of service provided to address of the need of the user. The choice is also impacted by the client's comprehension of the capacity of the various degrees of function of the different levels of health facilities. In Nigeria, health care structure is a framework consisting of both public and private health services. In the public segment, services include: Primary Health Care (PHC), Secondary and Tertiary health care which correspond to the different levels of administration. More so, in the private sector, the health care service utilization is capital intensive and health care service utilization is highly tied to monetary terms, and services are only rendered to those who patronize them.

Everywhere in the world, individuals with low financial capacity and socio-economic status are exposed to and defenseless against a great deal of health risks. This has prompted this research to determine carefully the relationship between income levels of people and the utilization of health care services in Calabar metropolis.

## 2 Literature review

### 2.1 Socio-economic differentials affecting health care services utilization

The socio-economic status of a nation will in all probability show evidence of its health conditions. Basically, good economy index signifies better health conditions and effective health care administrations and utilization among the people. According to Buor (2005), using developing countries as a case study (Nigeria inclusive), the low budgetary disbursement on health care services has negatively influenced the supply of adequate and effective health facilities and services. Likewise, poor economic status makes quality health care out of reach to a larger segment of the population which has influenced the effective health care services usage among individuals in urban cities. In view of these, Buor (2005) argues that, economic conditions that relate to the environment, as well as lifestyle and access to health care services are seen as important factors affecting people in obtaining adequate health care services utilization.

According to the World Health Organization (1999), nation's economic situation is perceived as influencing the provision and accessibility of services which transcend an individual's capacity to pay. It tends to affect effective healthcare services usage of such person or individual. It has been identified by Simkhada et al. (2008) that high charge/bills is also a strong factor that discourages individuals from utilizing health care services and facilities among low income earners in metropolitan cities.

According to Abang et al. (2016) the above issues have led to the preference of indigenous treatment and health care over modern medicine. Indigenous treatment and services charges are very moderate and accessible, in some cases, the users of this traditional medicine have access to credit facilities, which in modern medicine is not the case because charges are usually standardized as given by the Council or government, which has over the years affected the effective use of healthcare services from those found in low socio-economic groups (Mekonnen and Mekonnen, 2009). Anderson (2005), emphasized the role of economic indices in the utilization of health care services, and see family income as playing key role in the healthcare services usage pattern of families.

Also, the patronage of medical or healthcare services/facilities has been affected by other socio-economic variables. As observed by Okafor (Olugbenga-Bello and Adebimpe (2010), government employees make use of modern health services more than self-employed individuals such as farmers, traders and craftsmen, and suggests that such differential in utilization can be accounted for by socio-economic factor. Furthermore, they observed the following variables as affecting health care services utilization of clients.

#### 2.1.1 Economic/financial factor

According to Abbott et al. (2011), economic or financial vulnerability influences people's decision and behavior towards expenditure on health care services, goods or activities that can affect their health and health care utilization patterns. This implies that individual with low income may not have good access to health care service and other basic necessities of life due to their income status.

#### 2.1.2 Culture/income

According to Olugbenga-Bello and Adebimpe (2010), culture and income influence health care services because of the belief system of a people. The cultural viewpoint on the utilization of health care services implies that health needs are greatly determined by the presence of physical disease as well as cultural views and interpretation of illness. That is the way people perceive a particular health problem and how/where they can seek help.

#### 2.1.3 Occupation

Simkhada et al. (2008), notes that occupational difference or characteristics of individual have a major effect on healthcare service utilization. This means that, individual with highly paid jobs when ill have the likelihood to access good medical service than low paying jobs. Example of this categorization of description can be seen among civil servants and public servant. That is, civil servant due to limited resources has low access to good medical service compared to politicians who are the major ruling class in the society.

### 2.2 Income level and the usage of healthcare services

Nigeria, a country with low-income, does not have the capacity to address the essential healthcare needs of its populace. There has been an increasing awareness of challenges of both urban and rural dwellers health issues and the necessity to make better the situation (Hamid et al., 2005). Availability of healthcare in sub-urban localities is made worse by issues like, inadequate basic health facilities, shortage of health practitioners, socio-economic and physical hindrances (Ricketts, 2009). The major explanation offered as reason for self-treatment in a study in some African societies like Zambia was that individuals were not buoyant monetarily enough to seek modern health care, due to high cost of the services and care in facilities, and long travel distance for appointments and subsequent return for provision of services required (Atkinson et al., 2005) to them, this is both time consuming and expensive.

Other contentions concerning this issue is the assumption that most individuals are knowledgeable enough about their health needs and can evaluate whether the health condition is life - threatening. Gotsadze, Bennet, Ranson, & Gzirishvili (2005) observe that though efficacy of care and quality of treatment was the most significant deliberation for utilization of healthcare facilities, cost of treatment was undoubtedly considered by lower socio-economic and lower income earners. In Guinea and Benin, it was found that even the lowest income groups would seek and utilize health services if a product or service was available, if there was easier accessibility. Those in the low income level in Sri Lanka were only able to patronize low cost private health care facilities (Akin and Hutchinson, 1999) believing that the quality of service rendered would be improved and satisfactory (Bellow, Bellow & Warren; 2011). In addition, some range of literature confirms that women involved in second rate occupations with lower enumerations and compensations in developed and developing countries suffer significant deprivation, stresses and health inequalities (Cooper, 2002). Individual income tends to be a more important determining factor of health (Mackenbach, 2002). Found in another study in Southeast Nigeria, the rural populace was unwilling to give money in return for healthcare and services in advance rather more likely to pay in divided portions over a time duration. The claim in the present investigation is that consistency of income may be a more suitable or independent variable and indicator, for utilization of health care services.

## 3 Method and materials

This study was designed to examine income as one of the socioeconomic differentials influencing health care service utilization among the people of Calabar metropolis, Cross River State- Nigeria. The study had specific objectives which preceded the two hypotheses used for the study. These hypotheses are as follows:

1. There is no significant relationship between income level of the people and health care utilization in Calabar metropolis.
2. There is no significant difference between occupational status and health care utilization in Calabar Metropolis.

Survey design was adopted for the study using questionnaire, interviews and Focus Group Discussions (FGDs) on selected respondents in the study area. The study area was Calabar Metropolis of Cross River State, Nigeria. Calabar Metropolis comprises of two Local Government Areas: Calabar Municipality (using University of Calabar Teaching Hospital) and Calabar South Local Government Area (using General Hospital). The simple random sampling technique was employed to select the sample of three hundred and sixty (360) respondents drawn from both health workers, in-patients and out-patients as well as residents of the study area was used for the study and formed the sample size. The three hundred and sixty respondents were selected for the study through a multi-stage sampling technique. In order to make a good randomization in the study, the study area was divided into two basic clusters to represent each local government area. In Calabar South Local government area, a sample of one hundred and ninety-five (195) respondents were randomly selected out of which (85) respondents were drawn from the health workers, in-patients/out-patients in the hospitals and (110) drawn from residents of the area. Also a sample of one hundred and sixty-five (165) respondents was selected for the study in Calabar Municipality. From the above, a sample of seventy-five (75) respondents was used to represent the target population in Calabar Municipality. Within a cluster, participants were made to pick from box with papers written on them “Yes” and “No”. All participants who picked “Yes” in all the clusters and consented were enrolled for the study. This was repeated to obtain all the required sample size for each cluster. Two trained research assistants who were health workers from the two Local Government Areas were used to administer the questionnaires and also conduct the interviews.

A 25-item questionnaire was used to get quantitative data having close ended questions divided into two (2) sections containing. Section ‘A’ covered demographic data of respondents such as sex, age, religion, education, occupation, health workers, marital status and income level, while section B contained eighteen (18) item questions which made use of Likert scale presentation in relationship to the variable under study. The Likert scale rating method was adopted to measure attitudinal response of respondents in relation to the phenomenon under study.

Four (4) sessions of focus group discussion (FGD) were organized with health care users and health workers in University of Calabar Teaching Hospital and General Hospital Calabar, using twelve participants per-session. The focus group was adopted in this research work in order to achieve more realistic value of the research findings.

Descriptive analysis on the socio-demographic characteristics of respondents were carried out and presented in tables while hypotheses were tested at P = 0.05 using Chi –square and one-way analysis of variance (ANOVA) statistical tools whose formula are presented below. The one-way analysis of variance was employed to investigate the difference in the level of income among respondents seeking health care service utilization among inhabitants of Calabar Metropolis of Cross River State.

The formula for Chi-square is given as:

X^2^ = chi-square

$${\text{X}}^{2}=\frac{\sum {\left(\text{O}-\text{E}\right)}^{2}}{\text{E}}$$

Of = Observed frequencies
Ef = Expected frequencies
∑ = Summation

Formula for ANOVA:

I. Ssi = ssB + SSw

Where SST = Total sum of squares.

SSB = Between group sum of squares
SSw = within group sum of squares

$$\text{II.}\;\text{F}=\text{ratio}=\frac{\text{MSB}}{\text{MSW}}$$

Where MSB = Between group mean squares.

MSw = within group mean squares

$$\;\text{MSB}=\;\frac{\text{SSB}}{\text{dfb}}=\;\frac{\text{SSB}}{\text{K}-\text{I}}$$

$$\;\text{MSW}=\;\frac{\text{SSW}}{\text{dfw}}=\;\frac{\text{SSW}}{\text{N}-\text{K}}$$

Where dfb = K–I and.

K = number of group
Dfw = N–K
N = number of observation in each group

## 4 Results

Table 1 showed the responses of respondents to the sub-scale on income of people and health care service utilization, with two options of either “YES or NO”. As presented in Table 1 most of the respondents either ticked ‘YES’ or ‘NO’ to all the five items in this subscale. For item 1 which seeks to examine whether people do not go to private hospital because their charges are high. From the item, about, 284 (78.90%) responded ‘Yes’ while 76 (20.10%) ticked ‘No’. this means that majority of the respondents had a positive perception on the high cost as a barrier for lack orthodox patronage. Item 2 which examine whether daily earnings cannot afford most medical bills in public hospitals, the result showed that 250 (66.67%) respondent positively by ticking ‘Yes’ while 120 (33.33%) had it rather negative by ticking ‘No’, item 3 examine if most people fail to go for medical diagnosis due to cost involved, the result showed that 250 (66.67%) ticked ‘Yes’ to strongly agree with the statement while, 120 (33.33%) responded ‘No’ to the question. From the finding in the table presented above, the result implies that the independent variable (level of income) affect the dependent variable (health care service utilization) (see Tables 2, 3).

**Table 1:** Responses on income level of respondents.

S/N	Statement	Yes	No
1.	We do not go to private hospital because their charges are high	284 (78.90%)	76 (20.10%)
2.	Our daily earnings cannot afford most medical bills in public hospitals	250 (66.67%)	120 (33.33%)
3.	Most people fail to go for medical diagnosis due to cost involved	250 (66.67%)	120 (33.33%)

∗The number of people who responded to each item are as indicated, the percentage are written in parenthesis.

**Table 2:** ANOVA Descriptive Analysis for occupational status and health care service utilization (N = 360).

	N	Mean	Std. Deviation
Employed	220	15.64	2.741
Unemployed	87	15.84	2.357
Students	29	18.14	3.662
Retired	24	18.79	3.349
Total	360	16.10	2.940

Decision Rule: From the result as presented in the Table 2 above, the calculated F-ratio of 14.964 was greater than the critical F-ratio of 3.04 with 3 degrees of freedom. This result implied that the null hypothesis which stated that, there is no significant difference between individual occupational status and health care service utilization was rejected while the alternate hypothesis was accepted. It therefore means that individual occupational status influences health care service utilization. The difference between these groups, was statistically significant, hence, a post hoc test was not performed for multiple comparison between the dependent variable.

**Table 3:** ANOVA Descriptive Analysis for level of income and utilization of Health care services (N = 360).

	N	Mean	Std. Deviation
Low income earners	136	15.40	2.606
Moderate income earners	183	16.34	2.735
High income earners	41	17.32	4.150
Total	360	16.10	2.940

Decision Rule: From the result as presented in the table above, the calculated F-ratio of 14.964 was greater than the critical F-ratio of 3.04 with 3 degrees of freedom. This result implied that the null hypothesis which stated that, there is no significant difference between individual occupational status and health care service utilization was rejected while the alternate hypothesis was accepted. It therefore means that individual occupational status influences health care service utilization. The difference between these groups, was significant, hence, a post hoc test was not performed for multiple comparison between the dependent variable.

From the result as presented in the table above, the calculated F-ratio of 8.241 was greater than the critical F-ratio of 3.04 with 2 degree of freedom, this result therefore implies that the null hypothesis which stated that, there is no significant influence of income level of people and health care service utilization was rejected while the alternate hypothesis was accepted. It therefore means that income level of people influences health care service utilization. The difference between these group was statistically significant, hence, a post hoc test was not performed for multiple comparison between the dependent variable. In view of these Anderson (2016) have highlighted the role of economic factors in the utilization of health services, which sees family income and individual income as playing the most pivotal role in a pattern of health services utilization of any family or individual in a given society (see Table 4).

**Table 4:** ANOVA Descriptive Analysis for level of income and utilization of Health care services (N = 360).

	N	Mean	Std. Deviation
Low income earners	136	15.40	2.606
Moderate income earners	183	16.34	2.735
High income earners	41	17.32	4.150
Total	360	16.10	2.940

Decision Rule: From the result as presented in the table above, the calculated F-ratio of 8.241 is greater than the critical F-ratio of 3.04 with 2 degree of freedom, this result therefore implies that the null hypothesis which states that, there is no significant influence of income level of people and health care service utilization was rejected while the alternate hypothesis was accepted. It therefore means that income level of people influences health care service utilization. The difference between these group, was statistically significant, hence, a post hoc test was not performed for multiple comparison between the dependent variable.

To test hypothesis 2, one-way analysis of variance was adopted to test this hypothesis – occupational status and Health care service utilization. Furthermore, occupational status was categorized into four levels (employed, unemployed, students and retired) while the dependent variable was Health care service utilization. From the result as presented in the table above, the calculated F-ratio of 14.964 is greater than the critical F-ratio of 3.04 with 3 degree of freedom, this result therefore implies that the null hypothesis which states that, there is no significant difference between individual occupational status and health care service utilization was rejected while the alternate hypothesis was accepted. It therefore means that individual occupational status influences health care service utilization. The difference between these group, was statistically significant, hence, a post hoc test was not performed for multiple comparison between the dependent variable. The findings were in line with Adebimpe (2010), who noted that occupational difference or characteristics of individual have a significant influence on health care service utilization. That is, individual with highly paid jobs when ill have the likelihood to access good medical service than low paying jobs. In light of the assumption above, Abang (2016), reiterated that occupational status enhances good access to medical services, and improves health and well-being.

## 5 Discussion

Income and economic status have been identified as part of the important indicators of service utilization and the degree to which income influence health-seeking (Hodge et al., 2016). Most times the choice of health care facility and services used become dependent on the cost compared to the generally recognized advantages. As indicated by Buor (2005), the capacity to make payment for services informs the utilization of a particular health services. In most cases, some may be ready to pay for services, but the resources to compensate for the exchange of services, may not be available. Indisputably, low income can be an obstacle to health seeking and health services utilization thereby engendering a great health care burden on individuals (Gotsadze et al., 2005). In Nigeria, findings from the study reveals that the primary health care system especially has been poorly utilized and diverse factors are identified as its causes including, income of individuals, occupational status, proximity to clients/patients, services affordability, behaviour and attitude of service providers, availability of equipment and trained service providers (Krieger et al., 1997). Generally, individuals in a low income group are bound to suffer ill health and encounter health imbalances. Corresponding relationship was observed between low education levels, low social status, where individuals are more exposed to factors that may promote ill-health (Mackenbach and Howden-Chapman, 2003). Most of the participants in the Focus group discussion, had a negative view on the influence of income on client choice of health care service utilization. The respondents reiterated that, the major reason for their heaviness to access orthodox medicine is the cost of medical diagnosis and drugs, hence they rather go for cheap traditional Medicare.

## 6 Conclusion

Health is a basic human need in every given society, the high level of its development in any society could be ascertained by the quality of health services made accessible to the population, equitable distribution of health care services and facilities across a given society. Individuals access to available health services is seen to be influenced greatly by income level and occupation status of individuals and poses threat to the actualization of Sustainable Development Goals and other important aspirations of both individuals and the state. The study has been able to establish a stimulating correlation between income level and the utilization of health services among the people of Calabar metropolis. Health care services utilization has also been revealed to be determined by people's desire to obtain care, their level of knowledge of their health need and the knowledge of care accessibility and most importantly their ability to pay for services rendered. From this survey, low income individuals have been categorized as those who are unable to obtain prompt or adequate or certain types of health care services due to cost.

## 7 Recommendations

Based on the findings of this study, the following recommendations were made:

1. Since income level of respondents is a major determinant of health care service utilization, a significant improvement on individual income earning will go a long way to improve the choice of client or patient health care delivery service in hospital of their choice.
2. Since occupational status of individual also affects their choice of medical service, an improvement on individual's level of income through entrepreneurial or skill development can help the choice of individual in accessing medical service when faced with any health problem.
3. There is need for the promotion of individual and community knowledge of their health needs through community education and enlightenment campaigns. By so doing, individuals will be able to determine whether they require health attention. In addition, community education will go a long way to provide information on available health products and services and how they can utilize them for maximum benefits.
4. Cost of health care services should be minimized by the government/policy makers and made affordable for effective and equal utilization.

## Declarations

### Author contribution statement

Esther Archibong: Conceived and designed the experiments; Performed the experiments; Analyzed and interpreted the data; Wrote the paper.

Glory Bassey, R. Eneji, Brown Isokon: Performed the experiments; Analyzed and interpreted the data.

### Funding statement

This research did not receive any specific grant from funding agencies in the public, commercial, or not-for-profit sectors.

### Competing interest statement

The authors declare no conflict of interest.

### Additional information

No additional information is available for this paper.
